# Failure to normalize lymphopenia following trauma is associated with increased mortality, independent of the leukocytosis pattern

**DOI:** 10.1186/cc11157

**Published:** 2012-01-20

**Authors:** Daithi S Heffernan, Sean F Monaghan, Rajan K Thakkar, Jason T Machan, William G Cioffi, Alfred Ayala

**Affiliations:** 1 Division of Surgical Research, Department of Surgery, Warren Alpert Medical School of Brown University, Rhode Island Hospital, Providence, Rhode Island, USA; 2 Division of Statistics, Department of Orthopedics, Warren Alpert Medical School of Brown University, Rhode Island Hospital, Providence, Rhode Island, USA

## Abstract

**Introduction:**

Following trauma and systemic inflammatory response syndrome (SIRS), the typical response is an elevation of the total complete blood count (CBC) and a reduction of the lymphocyte count. This leukocytosis typically returns to normal within 48 hours. The persistence of a leukocytosis following trauma is associated with adverse outcomes. Although lymphocyte anergy and dysfunction following trauma is associated with increased risk for infection and sepsis, there is a paucity of data regarding the impact of a persistence of a low lymphocyte count in trauma patients.

**Methods:**

This is a retrospective review of prospectively collected data from trauma patients collected over the 5 years of September 2003 to September 2008. Patients were included if the injury severity score (ISS) was >/=15, and they survived at least 3 days. Demographic data, mechanism and injury severity score, mortality, and length of stay were collected from the medical record. Laboratory values for the first 4 hospital days were collected. Leukocyte, neutrophil and lymphocyte counts were extracted from the daily complete blood count (CBC). Patients were then grouped based on response (elevation/depression) of each component of the CBC, and their return, or failure thereof, to normal. Proportional hazards regression with time-varying covariates as well as Kaplan-Meier curves were used to predict risk of death, time to death and time to healthy discharge based on fluctuations of the individual components of the CBC.

**Results:**

There were 2448 patients admitted over the 5 years included in the analysis. When adjusting for age, gender and ISS the relative risk of death was elevated with a persistent leukocytosis (2.501 (95% CI = 1.477-4.235)) or failure to normalize lymphopenia (1.639 (95% CI = 10.17-2.643)) within the first 4 days following admission. Similar results were seen when Kaplan-Meier curves were created. Persistent lymphopenia was associated with shortest time to death. Paradoxically in survivors persistent lymphopenia was associated with the shortest time to discharge.

**Conclusions:**

Persistently abnormal CBC responses are associated with a higher mortality following trauma. This is the first report noting that a failure to normalize lymphopenia in severely injured patients is associated with significantly higher mortality.

## Introduction

Trauma remains the 5^th ^leading cause of death. Among survivors, significant morbidity occurs following hospitalization. Death from trauma follows a tri-modal distribution; first, at the scene of the trauma, second in the first 24-48 hours from devastating head injuries or massive blood loss and third with delayed deaths from multi-organ failure and sepsis[1-3]. Patients who do not succumb to early death, but who are not well enough to be discharged early, enter this third phase of trauma, a phase that is often fraught with infections (urinary tract infections, central line infections), debilitation and chronic wound management. Some patients may die during this phase of hospitalization. Following trauma a tremendous inflammatory response occurs (Systemic Inflammatory Response Syndrome - SIRS[4]) and almost simultaneously, an appropriate and extremely important counter inflammatory response is initiated (Compensatory Anti-inflammatory Response Syndrome -CARS[5]). Persistence of this inflammatory response has been associated with multi-organ failure. Disturbances in this inflammatory pattern such as excessive inflammation, persistence of inflammation, or a lack of the counter-inflammatory response, are believed to be associated with worse outcomes[5,6]. Gaining insight into the factors that lead to this prolonged immune and inflammatory dysfunction may offer therapy that would shorten hospital stay and help to alleviate the morbidity seen in this patient population.

SIRS is a well-defined occurrence following major trauma[4,7]. Both the leukocytosis and the neutrophil component of the SIRS response are also understood. However, trauma causes activation of all parts of the immune and inflammatory response. In this regard, there is a paucity of clinical literature pertaining to the other components of the white cell count as it relates to trauma. Lymphocytic loss and dysfunction is a well-documented finding in animal models of both SIRS and sepsis[8]. Preventing lymphocyte dysfunction, specifically preventing lymphocyte apoptosis following sepsis has been shown to improve mortality following sepsis[9]. The impact of the lymphocyte dysfunction in murine models is independent of the other components of the immune system. Specifically, we are interested in identifying the pattern of lymphocyte response to trauma, and whether lymphocyte profile was predictive of outcomes following traumatic injuries.

We hypothesize that the typical pattern for patients following a traumatic event is to develop lymphopenia shortly after the trauma, followed by rapid recovery to normal within the first 72 to 96 hours, and that deviation from this pattern would be associated with worsened outcomes.

## Materials and methods

This is a retrospective review of prospectively collected data from the trauma registry of Rhode Island's only Level 1 trauma center over the 5 year period of September 2003 to September 2008. Patients were included if they were moderately or severely injured, as denoted by an Injury Severity Score (ISS) of >/=15, aged 18 years or older, and they survived at least 3 days. Only patients with at least 2 complete blood counts over the 4 days to include all components (white cell count, lymphocyte count and neutrophil count) were included. Further, patients were excluded if they had non-survivable injuries as denoted by an ISS = 75. Patients with minimal injuries often do not display an exaggerated immune response, hence the exclusion of patients with ISS < 15. Clinical information obtained included age, gender, mechanism of trauma. Degree of injury was assessed by extracting the Injury Severity Score (ISS) and Trauma & Injury Severity Score (TRISS). Outcomes data included mortality at 21 days, total length of stay, of those who died within the 21 days - then time to death, and, of the survivors, time to healthy discharge.

The data for the Complete Blood Count (CBC) was obtained from the labs that were drawn as standard of care. Blood tests that were ordered by the trauma team were analyzed in standard fashion in the clinical laboratories of Rhode Island Hospital. Every Complete Blood Cell count (CBC) that was obtained for the first 4 days (96 hours from admission) on these patients was extracted from the medical record. This was further broken into the components of neutrophil count (percentage and absolute number) and lymphocyte count (percentage and absolute count). Since this was clinical data obtained during the normal course of patient care, the ranges for normal leukocytes, neutrophils and lymphocytes are those established by our clinical laboratory at Rhode Island Hospital. Specifically, normal white cell count = 4.0 - 11.0 × 10^9^/L, and leukocytosis was defined as any value above 11.0 × 10^9^/L. A normal neutrophil count =1.5 - 7.5 × 10^9^/L, and an elevated neutrophil count was defined as any value above 7.5 × 10^9^/L; normal lymphocyte count =1.0 - 4.0 × 10^9^/L and lymphopenia was defined as any value below 1.0 × 10^9^/L.

The white cell count was then segregated into three groups. The expected pattern following trauma is to develop an elevated white cell count which returns to normal within the first 4 days following the trauma (Group 1[leukocytosis = 1; Back to normal = 1]). Group 2 consisted of patients who developed a leukocytosis which never returned to normal within the first 4 days of the hospital stay (leukocytosis = 1; Back to normal = 0). Group 3 consisted of patients who did not develop a leukocytosis within the first 4 days following the trauma. A similar set of groupings was undertaken for the neutrophil count, namely Group 1 = elevated neutrophil count, returned to normal, Group 2 = elevated neutrophil count, never returned to normal, and Group 3 = never developed an elevated neutrophil count.

Next we divided the patients by lymphocyte count. The normal pattern following trauma is for patients to undergo a loss of lymphocytes, and then to recover shortly thereafter. Therefore this led to the formation of 3 groups for the purpose of the analysis. Group 1 (Ever Lymphopenic = 1; Back to Normal = 1) was the expected pattern, whereby there was a loss of lymphocytes following trauma, which returned to normal within the first 4 days. Group 2 (Ever Lymphopenic = 1; Back to Normal = 0) included patients who displayed a lymphopenia shortly after presentation, but whose lymphocyte count did not return to normal over the first 4 days. Group 3 (Ever Lymphopenic = 0; Back to Normal = 0) consisted of patients who maintained a normal lymphocyte count, and did not at any point develop lymphopenia throughout the first 4 days of the hospitalization.

Each of the three divisions, namely White cell count fluctuations, neutrophil count fluctuations and thirdly lymphocyte fluctuations were reviewed independently of each other. This allowed association of each cell type trend with outcomes independent of changes in the other components. Rhode Island Hospital Institutional Review Board approved this study. No patient consent was needed.

### Statistical analysis

Proportional hazards regression with time-varying covariates was used to predict time risk of death at 21 days based the groupings of leukocytes, neutrophils, and lymphocytes described above. This analysis was done adjusting for age gender, ISS, and the interaction between leukocyte group and time to death. To assess the impact upon mortality of the variation in leukocytes, neutrophils, and lymphocytes Kaplan-Meier curves were created. We then assessed the impact of lymphocyte patterns upon time to death, and, among the survivors, the time to healthy discharge. Thus, Kaplan-Meier curves were created for time to death comparing across each of the lymphocyte groups. We also analyzed Kaplan-Meier curves for the three lymphocyte groups with respect to time to healthy discharge by excluding all patients who died at any time during the hospitalization.

## Results

Overall, there were 2,448 patients with ISS >/=15, but not 75, who survived at least three days over the five year study period (table 1). The mean ISS was 22.9 (+/- 7.9). 1,689 patients (69%) were males, with a mean age of 52.5 years. 94% of patients sustained blunt mechanism of trauma, with the top 2 leading causes of trauma were motor vehicle collision and falls. The mean ICU length of stay was 3.9 days (+/- 10.3) and the mean hospital length of stay was 13.1 days (+/- 22). Of those who were mechanically ventilated for more than 24 hours, the mean ventilator days was 7.8 (+/- 12.2). The overall mortality rate was 20.5% (504 patients) (table 1).

**Table 1:** Overall characteristics of the patient population

Factor	
Age (years)	52.5 (+/- 23.14)
Male Gender	1,689 (69%)
Blunt mechanism of trauma	2,301 (94%)
ISS	22.9 (+/- 7.8)
TRISS	10.5 (+/- 2.7)
Hospital Length of Stay (days)	13.1 (+/- 22)
ICU Length of Stay (days)	3.9 (+/- 10.3)
Ventilator days	7.8 (+/- 12.2)

Reported as mean +/- standard deviation. ISS = Injury Severity Score. TRISS = Trauma & Injury Severity Score.

Proportional hazards regression, adjusting for age, gender and ISS to predict death with relation to the fluctuations in each of the components of the white blood cell count are displayed in table 2. A return of leukocytosis to normal within the first 4 days was protective against death (RR = 0.420; 95% CI = 0.216-0.816). A persistence of the leukocytosis was associated with a 2.501 fold increase in risk of death (95% CI = 1.477-4.235). There was no significant association between risk of death and either a neutrophila which returned to normal (RR = 0.668; 95% CI = 0.39-1.144) or a persistent neutrophila (RR = 1.183; 95% CI = 0.784-1.784). A lymphopenia which returned to normal within the first 4 days was associated with a decreased relative risk of death (RR = 0.497; 95% CI0.27-0.916). However, a lymphopenia which failed to return to normal was associated with a 1.64 fold increase in relative risk of death (95% CI = 1.017-2.643) (table 2).

**Table 2:** Adjusted Risk of Death for each group based upon pattern of each component of the CBC and its interaction with time to death (survival to 480 hours)

*Factor*		*Increase relative risk of death*	*95% Confidence* *Interval*	*p value*
Leukocytosis	Return to normal	0.420	0.216 - 0.816	0.004
	Remains elevated	2.501	1.477 - 4.235	< 0.001
Neutrophilia	Return to normal	0.668	0.390 - 1.144	0.7
	Remains elevated	1.183	0.784 - 1.784	0.3
Lymphopenia	Return to normal	0.497	0.270 - 0.916	< 0.001
	Remains low	1.639	1.017 - 2.643	0.01

**Figure 1:**
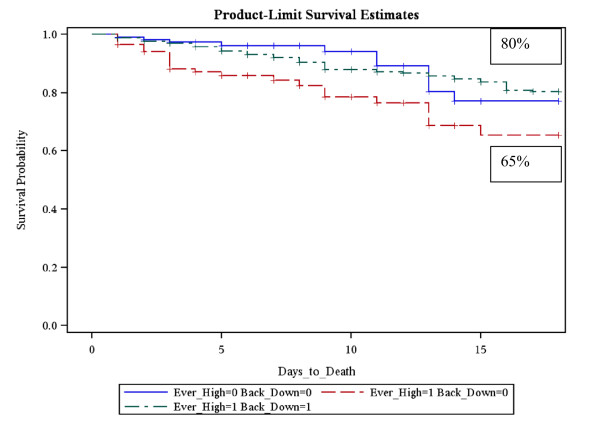
**Kaplan-Meier survival graph with respect to the leukocyte groupings**. Patients who developed a leukocytosis which never returned to normal had the lowest probability of survival (65% survival rate at 21 days). **Group 1 = (green dashed line) **Ever Leukocytosis = 1; Back to Normal = 1; **80% 21 day survival, Group 2 = (red dashed line) **Ever Leukocytosis = 1; Back to Normal = 0; **65% 21 day survival, Group 3 = (blue dashed line) **Ever Leukocytosis = 0; Back to Normal = 0; **77% 21 day survival**.

The data was then analyzed for the probability of survival over time using Kaplan-Meier curves for the specific fluctuations of the individual components of the white blood cell count, namely leukocytes, neutrophils and lymphocytes. Initially Kaplan-Meier curves were drawn for probability of survival with respect to leukocyte fluctuations (Figure1). Patients who developed a leukocytosis which did not return to normal within the first 4 days displayed the lowest probability of survival of 65% at 21 days when compared with either patients who developed leukocytosis which did return to normal with 80% 21 day survival (p = 0.0085) or patients who never developed leukocytosis with 77% 21 day survival (p = 0.003). There was no significant difference in 21 day survival between patients who developed leukocytosis which returned to normal within 4 days compared with patients who never developed a leukocytosis (p = 0.99).

**Figure 2:**
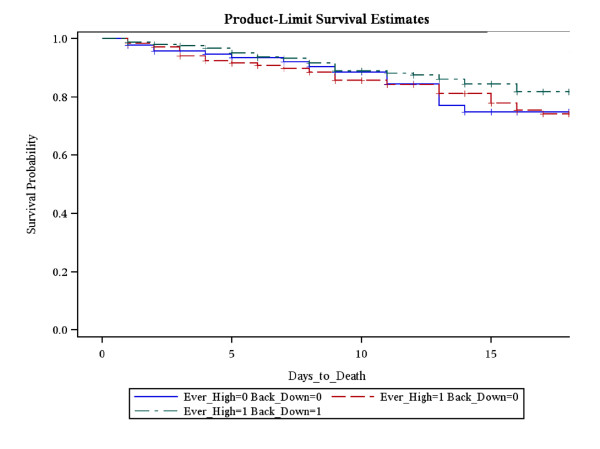
**Kaplan-Meier survival curve with respect to the pattern of the neutrophil fluctuations**. There was no difference in survival comparing the 3 patterns, namely elevated-returned to normal, elevated-stayed elevated and never elevated. **Group 1 = (green dashed line) **Ever Neutrophilia = 1; Back to Normal = 1; **81.2% 21 day survival, Group 2 = (red dashed line) **Ever Leukocytosis = 1; Back to Normal = 0; **81.8% 21 day survival, Group 3 = (blue dashed line) **Ever Leukocytosis = 0; Back to Normal = 0; **74.7% 21 day survival**.

Next this was repeated for probability of survival by neutrophil groupings (Figure 2). There was no difference in probability of 21 day survival between patients who never developed a neutrophilia (74.7%) either compared with patients who developed a neutrophilia which remained elevated over the first 4 days (74.3%; p = 0.99) or patients who developed a neutrophilia which returned to normal within 4 days (81.8%; p = 0.4). Furthermore, there was no difference in 21 day survival comparing patients who developed a neutrophilia that remained elevated compared with those whose neutrophilia resolved (p = 0.24)

**Figure 3:**
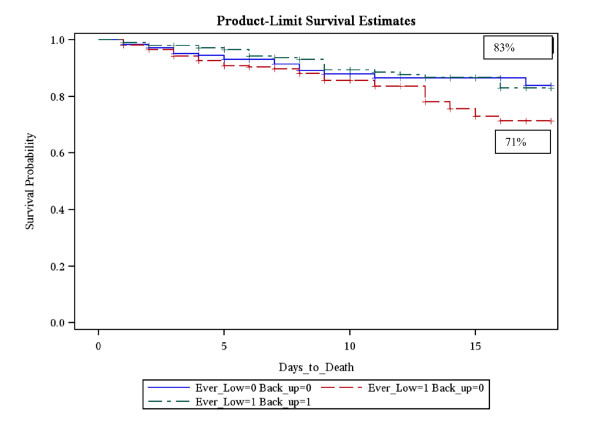
**Kaplan-Meier survival graph with respect to the Lymphocyte groupings**. Patients who displayed lymphocyte loss with no subsequent recover of Lymphocyte number had the lowest survival of 71%. **Group 1 = (green dashed line) **Ever Lymphopenic = 1; Back to Normal = 1; **83% 21 day survival, Group 2 = (red dashed line) **Ever Lymphopenic = 1; Back to Normal = 0; **71.3% 21 day survival, Group 3 = (blue dashed line) **Ever Lymphopenic = 0; Back to Normal = 0; **83.75% 21 day survival**.

Within the lymphocyte subdivisions, there were 792 patients (32.4%) in Group1 (lymphopenia - returned to normal), 817 patients (33.4%) in Group 2 (lymphopenia - persistent lymphopenia) and 839 patients (34.2%) in Group 3 - (never lymphopenic). With respect to the lymphocyte patterns, there was no difference in 21 day probability of survival between patients who displayed the expected pattern - Group 1, lymphocyte loss followed by recovery, and Group 3, patients who never exhibited a lymphocyte loss (83% versus 83.75%; p = 1.0). Patients in Group 2, who displayed lymphocyte loss, which did not recover within the first 4 days, had the lowest probability of survival at 21 days at 71.3% (p = 0.037) (Figure 3).

**Figure 4:**
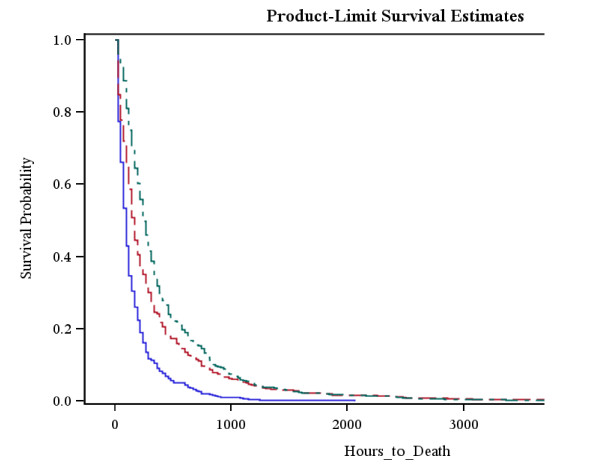
**Time to Death Curves**. Patients who were never Lymphopenic displayed the shortest average time to death. Patients with lymphopenia which returned to normal in the first 4 days displayed the longest average time to death. **Group 1 = (green dashed line) **Ever Lymphopenic = 1; Back to Normal = 1; **Group 2 = (red dashed line) **Ever Lymphopenic = 1; Back to Normal = 0; **Group 3 = (blue dashed line) **Ever Lymphopenic = 0; Back to Normal = 0;

We then analyzed the time to death with respect to lymphocyte pattern in the group of 504 patients who died (Figure 4). Average time to death was shortest in Group 3 (never lymphopenic) at 28.2 days, followed by Group 2 (persistent lymphopenia) at 32.5 days and was longest for patients who died in Group 1(lymphocyte loss returned to normal) at 37.5 days (p < 0.0001).

**Figure 5:**
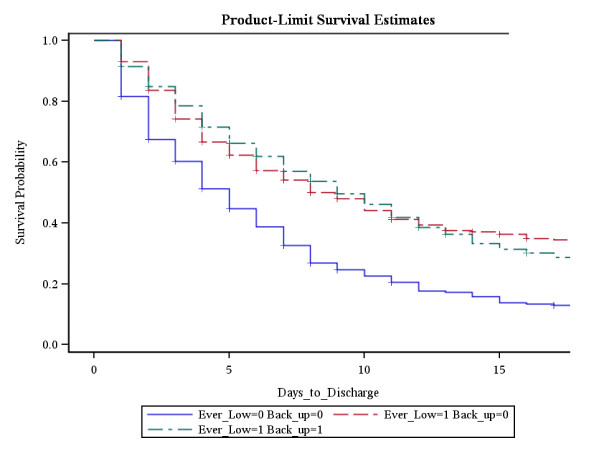
**Time to discharge**. Of the survivors, patients in whom lymphocytes did not fall low had the shortest quickest time to discharge. There was no difference between the other 2 groups. **Group 1 = (green dashed line) **Ever Lymphopenic = 1; Back to Normal = 1; **Group 2 = (red dashed line) **Ever Lymphopenic = 1; Back to Normal = 0; **Group 3 = (blue dashed line) **Ever Lymphopenic = 0; Back to Normal = 0;

The total population was then censored to exclude any patient who died while in hospital. This then left us with the "All-alive" group constituting 1,944 patients. We analyzed this "all-alive" group to assess the time to discharge (Figure 5), using Kaplan-Meier analysis. The time to discharge was shortest among Group 3 (never lymphopenic) throughout all time points compared with both Group 1 (lymphopenia returned to normal) and Group 2(persistent lymphopenia) (p < 0.0001). There was no difference in time to discharge between group 1 (lymphopenia returned to normal) and group 2 (persistent lymphopenia).

## Discussion

Trauma remains the 5^th ^leading cause of death in the United States, and is rapidly been understood as a leading cause of both mortality and significant morbidity world-wide. Devastating head injury and non-salvageable blood loss remain the leading causes of death in early time points following admission for trauma. Easily measurable and trackable parameters are essential to aid clinicians to appreciate the degree of stress and SIRS response in critically ill and injured patients following poly-trauma, shock and subsequent organ dysfunction.

The leukocytosis response following trauma is well described. A SIRS response comprises of leukocytosis which rapidly returns to normal over the initial 3 days. The neutrophil pattern is also well recognized and they clearly play an important role in the response to tissue injury[10]. It is well recognized that a perpetuation of the SIRS response is associated with worse outcomes, specifically as they relate to infectious complications, in trauma patients[11].

Several animal models have shown either lymphocyte loss or lymphocyte dysfunction following various models of injury, namely acute lung injury, sepsis or burns[12-14]. Lymphocytes, separate from the other components of the leukocyte population, have been demonstrated to be direct contributors to acute lung injury[15]. Lymphocytic anergy has been seen in humans following both trauma[16,17] and sepsis[18]. However, there is a paucity of human data in the literature addressing the impact of lymphocyte fluctuations and the progression of lymphocyte numbers in the early phase following trauma as it relates to mortality. We believe that our work adds clinical evidence confirming the murine data[6].

To best assess the effects of trauma on the inflammatory response, we chose patients who were most likely to have sustained an inflammatory response, namely patients with moderate to severe injuries as denoted by an Injury Severity Score of >/=15. To minimize the effects of those early deaths, we excluded patients with an ISS of 75 (non-survivable) and included only patients who survived at least 3 days.

We noted that leukocytosis and specifically a persistence of leukocytosis was associated with a 2.5-fold increase in mortality. This is in keeping with previous publications as well as our understanding of the detrimental effect of a prolonged inflammatory response[11]. When the lymphocytic response was reviewed, it was intriguing that one third of patients never developed a lymphopenia. Although statistically significant, the clinical differences in age, gender and Injury severity in this Group 3 (never lymphopenic) were small.

The mortality was highest in patients who developed a lymphopenia that did not recover, reflecting the concept that recovery from lymphocyte loss is essential to surviving traumatic injuries. Interestingly, the time to both death and discharge was shortest in Group 3 (never lymphopenic), from which we may infer that patients who do not display a lymphocyte loss are going to die earlier from their injuries, but that if they do not die early, then these patients will recover quicker and be discharged sooner. We postulate that patients who do not exhibit an appropriate lymphocyte response to the initial trauma, are incapable of tolerating the ensuing stress response and secondary insults, such as pneumonia, that are often seen in these moderate to severely injured trauma patients. Future work will attempt to address associations between lymphocyte profiles and secondary events such as pneumoniae.

In a study of 105 patients, Cheadle et al described a significant lymphopenia, which was noted to be maximal at 3 days and was noted to recover over the study period[19]. The lymphocytes, and several subsets, were lowest in patients who died (*n *= 15) or developed severe infections. Our study expands on this concept to a larger study population, allowing us to address the best profile of lymphocyte response to trauma. Akin to their data, we have demonstrated a significant association between a lack of lymphopenia, or a failure to resolve lymphopenia, on mortality.

Yamada et al[20] in a large animal model demonstrated that lymphopenia occurred with a laparotomy. Further, this lymphopenia occurred via stress-induced apoptosis. Clearly the effect of stress on the lymphocytes occurs early following the response. In a study of 20 patients, Rainer et al[21] demonstrated lymphopenia in all but one within 6 hours following presentation after the trauma. Their study was limited to blood draws within the first 24 hours, and was not powered to assess trends of the lymphocyte and how these may relate to the subsequent hospital course. Similar to this observation, over 65% of our patient population exhibited a lymphopenia. Our study takes the observation of Rainer to the next level with a combination of a significantly larger population as well as extending the time period of lymphocyte tracking over the first 4 days following the trauma.

Zahorec noted an association between the severity of illness and the degree of both lymphopenia and neutrophilia in 90 ICU patients[22]. Patients with the highest SOFA and APACHE II were noted to have both the highest neutrophilia and the lowest lymphopenia within the first 24 hours of ICU admission. However, the ssociation was based on the ratio of neutrophils to lymphocytes (the neutrophil/lymphocyte stress factor) rather than an independent association with the absolute lymphocyte count. Further concerns with this study included the mixture of patient population, medical elective surgical and septic, as well as a small population, only 28 of the 90 presented with acute critical illness. Our study, for the first time clearly demonstrates that the profile of the absolute lymphocyte number was independent of the remainder of the CBC profile. Furthermore, our population consists of a relatively homogenous population, namely trauma patients.

Rather than just passive bystanders in the inflammatory and immune response to trauma, lymphocytes play a critical key role in the proper modulation of the inflammatory response, and the lymphopenia seen is a consumption and activation of these cells. A perpetuation of this lymphocyte loss generally reflects cellular exhaustion, desensitization and down-regulation of the lymphocytes, and specifically certain subsets[23]. This exhaustion of the lymphocytic response may explain why patients who fail to recover their lymphopenia following trauma have the lowest associated survival (73% versus 83%).

Interestingly, patients who did not display any component of the typical response, namely never lymphopenic, had both the shortest time to death among those who died, as well as the shortest time to discharge among survivors. A lack of immune fluctuations may allow patients to rapidly recover from the traumatic injuries that do not require prolonged in-hospital treatment, and thus resolve their inflammation and be suitable for early discharge. Patients who have ongoing stress and need for in-hospital treatments they may fail to have a robust immune response to tolerate the ongoing management of the traumatic injuries. This may reflect the concept that some inflammatory response is beneficial, but no immune response is detrimental. Moderately and severely injured trauma patients often have ongoing stress responses due to either repeat operative intervention due to cumulative nature of their injuries, or due to 2^nd ^hit phenonomena such as infection. We believe that this dampened inflammatory response (never lymphopenic) reflects an inability to display an appropriate inflammatory response (due to lymphocyte exhaustion) in relation to ongoing stress. Tracking the immune response, and particularly the lymphocyte profile over the early course of a patients stay, namely the first 4 days, may allow us to predict which patients may not survive these 2^nd ^hit episodes.

There are several limitations of this study. First, the data addresses association with death and other outcomes, and is not a direct statement of effect. Further, the data is limited by the inability to address causality of death. However, we attempted to exclude causes of death which are potentially non-survivable, such as massive hemorrhage or devastation head injuries, by excluding patients who died within the first 72 hours, as well as excluding any patient with an ISS of 75 (lethal injury). Furthermore, this is a retrospective review of prospectively collected data and not all patients had continuous laboratory draws over the 4 days. The analysis is based on the assumption that all patients are normal prior to the traumatic event. This is necessary since traumatic injuries are not elective or planned. Laboratory draws were dictated by the primary treating team, and thus we were not able to analyze specific time points association. However, the purpose of the study was address whether failure to recover from lymphopenia within 4 days was associated with mortality, rather than any association between time to lymphopenia or time recovery and outcome. Future expansion of this work will be to attempt to abstract infectious complications and possible causes of death in this population.

There are several strengths to this current study. Here the leukocyte response was followed over a prolonged time frame of 4 days, rather than a single time point. A further strength of the study was our use of absolute lymphocyte numbers and not just percentages as well as analyzing all components of the white cell count together. Studies that have reported patient lymphocyte percentages[17,24] without the absolute numbers, or without comparing or stratifying lymphocyte to neutrophil counts, cannot ascertain to what extent their findings are based on the neutrophil component and to whether or not their lymphocyte findings are truly independent.

## Conclusions

Our data supports the animal model literature which shows that lymphocytes play a key role in the immune response to trauma and that this lymphocytic response is independent of the often cited neutrophil response. Patients who either failed to develop lymphopenia, or who failed to appropriately resolve lymphopenia were associated with significantly higher mortality and shorter time to death. This early information may better help the physician identify those critically ill patients who are at greatest risk of developing a morbid condition, thereby allowing the design of more realistic treatment plans for these individuals.

## Key messages

• SIRS response following traumatic injury is associated with lymphocyte loss

• A persistently low lymphocyte level over the first 4 days following moderate and severe injury is associated with increased mortality and increased hospital length of stay

• The association between failure to normalize lymphocyte number with worsening outcomes is suggestive of a key role for lymphocytes in the response to traumatic injuries

• The lymphocyte profile, and the association with outcomes is independent of the rest of the leukocyte response to trauma

## Abbreviations

95% CI: 95% confidence intervals; CARS: compensatory anti-inflammatory response syndrome; CBC: complete blood count; ICU: intensive care unit; ISS: injury severity score; RR: relative risk; SIRS: systemic inflammatory response syndrome.

## Competing interests

The authors declare that they have no competing interests.

## Authors' contributions

JTM was responsible for statistical assistance and data analysis. WGC was responsible for manuscript drafting and revision for intellectual content. AA and DSH were responsible for concept and design, data acquisition and analysis, manuscript drafting and revision for intellectual content. RKT was responsible for concept and design and data acquisition and analysis. SFM responsible for concept and design, data acquisition and analysis, statistical assistance and data analysis, manuscript drafting and revision for intellectual content. All authors have approved the final manuscript for publication.
